# Diagnosis of Cardiac Amyloidosis on Echocardiography Using Artificial Intelligence

**DOI:** 10.1161/CIRCIMAGING.125.018991

**Published:** 2026-02-12

**Authors:** Adam Ioannou, Michel G. Khouri, Takeshi Kitai, Sreekanth Vemulapalli, Chung-Lieh Hung, Sze Chi Lim, Matthew Frost, Weile Wayne Tee, Josephine Mansell, Awais Sheikh, Lucia Venneri, Yousuf Razvi, Aldostefano Porcari, Ana Martinez-Naharro, Muhammad U. Rauf, Helen Lachmann, Philip N. Hawkins, Ashutosh Wechelakar, William Moody, Francesco Bandera, Justin A. Ezekowitz, Carolyn S.P. Lam, Scott D. Solomon, Julian D. Gillmore, Marianna Fontana

**Affiliations:** 1 National Amyloidosis Centre, University College London, Royal Free Campus, London, United Kingdom (A.I., J.M., A.S., L.V., Y.R., A.P., A.M.-N., M.U.R., H.L., P.N.H., A.W., J.D.G., M. Fontana).; 2 Duke University Health System, Durham, NC (M.G.K.).; 3 Department of Heart Failure and Transplantation, National Cerebral and Cardiovascular Centre, Osaka, Japan (T.K.).; 4 Duke Clinical Research Institute, Durham, NC (S.V.).; 5 Institute of Biomedical Sciences, Mackay Medical College, Taipei, Taiwan (C.-L.H.).; 6 Us2.ai, Singapore (S.C.L., M. Frost, W.W.T.).; 7 Department of Cardiology, Queen Elizabeth Hospital, Birmingham, United Kingdom (W.M.).; 8 Institute of Cardiovascular Sciences, University of Birmingham, Edgbaston, United Kingdom (W.M.).; 9 Heart Failure and Rehabilitation Unit, IRCCS MultiMedica, Sesto San Giovanni, Milan, Italy (F.B.).; 10 Department for Biomedical Sciences for Health, University of Milano, Italy (F.B.).; 11 University of Alberta, Edmonton, Canada (J.A.E.).; 12 National Heart Centre Singapore & Duke-National University of Singapore (C.S.P.L.).; 13 Brigham and Women’s Hospital, Harvard Medical School, Boston, MA (S.D.S.).

**Keywords:** amyloidosis, artificial intelligence, deep learning, echocardiography, phenotype

## Abstract

**BACKGROUND:**

Diagnosing cardiac amyloidosis (CA) on echocardiography can be challenging due to the imaging overlap between CA and more prevalent causes of a hypertrophic phenotype. This study sought to (1) evaluate the performance of artificial-intelligence (AI) derived measurements incorporated into the established multiparametric echocardiographic scoring system to detect CA; (2) develop and validate an AI-based deep-learning model for video-based detection of CA on echocardiography.

**METHODS:**

The study population comprised 5776 patients (CA, 2756; controls, 3020). The training data set included patients from the UK National Amyloidosis Center and Taiwan MacKay Memorial Hospital (CA, 2241; controls, 2130). External test data sets were obtained from the US Duke University Health System (CA, 334; left ventricular hypertrophy controls, 668) and Japan National Cerebral and Cardiovascular Center (CA, 181; left ventricular hypertrophy controls, 222).

**RESULTS:**

The multiparametric echocardiographic score computed using AI-derived measurements achieved an accuracy of 79.5% (sensitivity, 75.4%; specificity, 81.5%) in the United States cohort and 79.7% (sensitivity, 81.6%; specificity, 78.1%) in the Japan cohort. The deep-learning model demonstrated accuracies of 96.2% (sensitivity, 96.8%; specificity, 95.7%) and 95.8% (sensitivity, 97.3%; specificity, 94.3%) in the internal validation and internal test sets, respectively. External validation of the deep-learning model showed accuracies of 87.5% (sensitivity, 86.6%; specificity, 87.9%) in the United States and 88.4% (sensitivity, 92.3%; specificity, 85.3%) in the Japanese cohort. Subgroup analysis demonstrated that the deep-learning model showed robust discrimination of CA from other hypertrophic phenocopies: CA versus hypertension (area under the curve [AUC], 0.92 [95% CI, 0.91–0.94]), CA versus hypertrophic cardiomyopathy (AUC, 0.91 [95% CI, 0.87–0.94]), CA versus aortic stenosis (AUC, 0.93 [95% CI, 0.90–0.95]), CA versus chronic kidney disease (AUC, 0.93 [95% CI, 0.91–0.95]). The deep-learning model was able to classify a greater proportion of patients compared with the AI-derived multiparametric echocardiographic score and achieved superior diagnostic accuracy (AUC, 0.93 [95% CI, 0.91–0.95] versus AUC, 0.88 [95% CI, 0.85–0.90]; *P*<0.001).

**CONCLUSIONS:**

Both the multiparametric echocardiographic score computed from AI-derived measurements and the fully automated deep-learning model can accurately identify patients with CA in globally diverse cohorts, with the deep-learning model providing superior performance.

**Clinical Perspective**

Cardiac amyloidosis is an underrecognized and underdiagnosed cardiomyopathy, which can be challenging to detect on echocardiography due to the imaging overlap with other causes of left ventricular hypertrophy. This international multicenter study demonstrates that both the multiparametric echocardiographic score computed from artificial-intelligence-derived measurements and the fully automated deep-learning model can accurately identify patients with cardiac amyloidosis across a range of health care settings. Both techniques could be integrated into clinical practice as fully automated background screening tools that are applied without any human interaction to echocardiograms to help raise the suspicion of cardiac amyloidosis. Such an approach has the potential to identify possible cases of cardiac amyloidosis, which may have otherwise been overlooked, and prompt a referral to a specialist center for confirmatory diagnostic tests and early initiation of disease-modifying therapies.

Cardiac amyloidosis (CA) is caused by the deposition of misfolded proteins in the form of amyloid fibrils within the myocardium, resulting in a progressive and ultimately fatal cardiomyopathy.^1,2^ The overwhelming majority of cases are due to the deposition of misfolded transthyretin (transthyretin amyloidosis [ATTR]) protein and misfolded immunoglobulin light-chain (light-chain amyloidosis) proteins.^3,4^ Despite improvements in cardiac imaging, CA remains an underrecognized and underdiagnosed entity, with many patients being diagnosed at a late stage with advanced cardiac disease. The advent of efficacious and specific disease-modifying therapies has significantly improved outcomes in patients with CA; however, early diagnosis and prompt initiation of disease-modifying therapies remain crucial.^5–7^

Echocardiography is the most widely accessible cardiac imaging modality and is uniquely positioned to characterize cardiac structure while simultaneously assessing systolic and diastolic function, and represents a crucial first-line investigation in patients with cardiac disease and suspected CA. Cardiac amyloid infiltration is characterized by biventricular wall thickening, impaired longitudinal systolic function, and impaired relaxation.^8^ Although recognition of these features can raise the suspicion of CA, they are often overlooked outside of specialist centers and may only be detected at a late disease stage.^2^ These diagnostic challenges are compounded by the significant overlap in echocardiographic findings between CA and other hypertrophic phenotypes. Diagnostic echocardiographic scoring systems harness a combination of metrics to improve diagnostic accuracy, but manual interpretation of echocardiograms remains time-consuming and requires highly trained specialists.^9^

Deep learning artificial-intelligence (AI) based algorithms have already shown great promise in cardiovascular imaging.^10,11^ Elimination of manual operator contouring enables AI to provide precise measurements of structural and functional parameters, while automated neural networks developed through deep learning have been shown to improve diagnostic accuracy across a wide range of cardiovascular diseases.^12–14^

This study sought to (1) evaluate the performance of AI-derived measurements incorporated into the established multiparametric echocardiographic scoring system to detect CA; (2) develop and validate an AI-based deep-learning model for video-based detection of CA on echocardiography.

## Methods

The data that support the findings of this study are restricted by the institutional ethics committees to protect patient privacy and, therefore, cannot be shared.

### Data Sources and Study Population

#### Training Data Sets

The model was developed using data from the National Amyloidosis Center (NAC, London, United Kingdom) and Taiwan MacKay Memorial Hospital (TMMH, Taipei, Taiwan). These sites were combined to produce a balanced cohort that comprised both CA cases and a diverse range of control cases.

The NAC echocardiography data set comprises transthoracic echocardiography images and reports from patients referred for suspected CA between 2007 and 2021. Ground-truth labels for both CA cases and controls were established through final clinical diagnoses from the reports. The cohort was unselected, meaning that all consecutive patients were included without exclusion criteria. Confirmed cases included monoclonal immunoglobulin light-chain CA and transthyretin CA. All patients underwent a comprehensive diagnostic work-up, including clinical evaluation, echocardiography, serum and urine biochemistry, including cardiac biomarkers, serum free light chain assay, and serum and urine immunofixation. Subsequent specialist investigations included cardiac magnetic resonance, bone scintigraphy, and histological examination. Transthyretin CA was diagnosed according to validated criteria, and light-chain CA was confirmed by central histological review, including Congo red staining. Cardiac involvement for all patients was established by cardiac magnetic resonance.^15^ This yielded 2241 patients with confirmed CA and 604 patients who tested negative for CA but had evidence of left ventricular hypertrophy (LVH) on echocardiography

The TMMH data set represents a real-world, community-based cohort identified between 2009 and 2021. Echocardiograms are conducted by a diverse group of operators, including general sonographic technicians and doctors, with varying skills and experience levels. The cohort was drawn from a large-scale, multi-cohort study on automated heart function and included patients with heart failure with reduced ejection fraction, hypertension, and healthy individuals. In the original study design, patients with conditions that introduce significant confounding structural changes, such as congenital heart disease, significant valvular disease, isolated right-sided heart failure, pulmonary hypertension, acute coronary syndrome, or end-stage renal disease, were excluded to create a well-defined heart failure cohort.^11^ As systematic screening for CA was not performed, a team of 4 certified sonographers thoroughly reviewed the TTEs to select controls. Based on imaging criteria alone, they identified 1526 patients with no apparent features of CA, following the 2019 Expert Consensus Recommendations for Multimodality Imaging in Cardiac Amyloidosis.^16^

In total, 10 146 apical 4-chamber (A4C) videos from 4371 patients were assembled for model training, validation, and internal testing. The data set was split into an 80-10-10 ratio such that 80% of the data goes to train the model, and the remaining is equally split for internal validation and testing.

#### External Validation Data Sets

Two independent data sets were used for external testing: Duke University Health System (DUHS, Durham, NC) and the National Cerebral and Cardiovascular Center (NCVC, Osaka, Japan). Patients from DUHS and NCVC were identified between 1995 and 2024 and 2019 to 2024, respectively. Both data sets included patients with CA based on established diagnostic criteria following a comprehensive diagnostic workup, and controls were patients without *International Classification of Diseases*-9/10 codes for CA and diagnosed with alternative causes of left ventricular hypertrophy, such as aortic stenosis, hypertrophic cardiomyopathy, end-stage kidney disease, and hypertension. Diagnoses of transthyretin CA and light-chain CA were established based on the same validated diagnostic criteria that were used at the NAC.^15^

**Figure 1.**
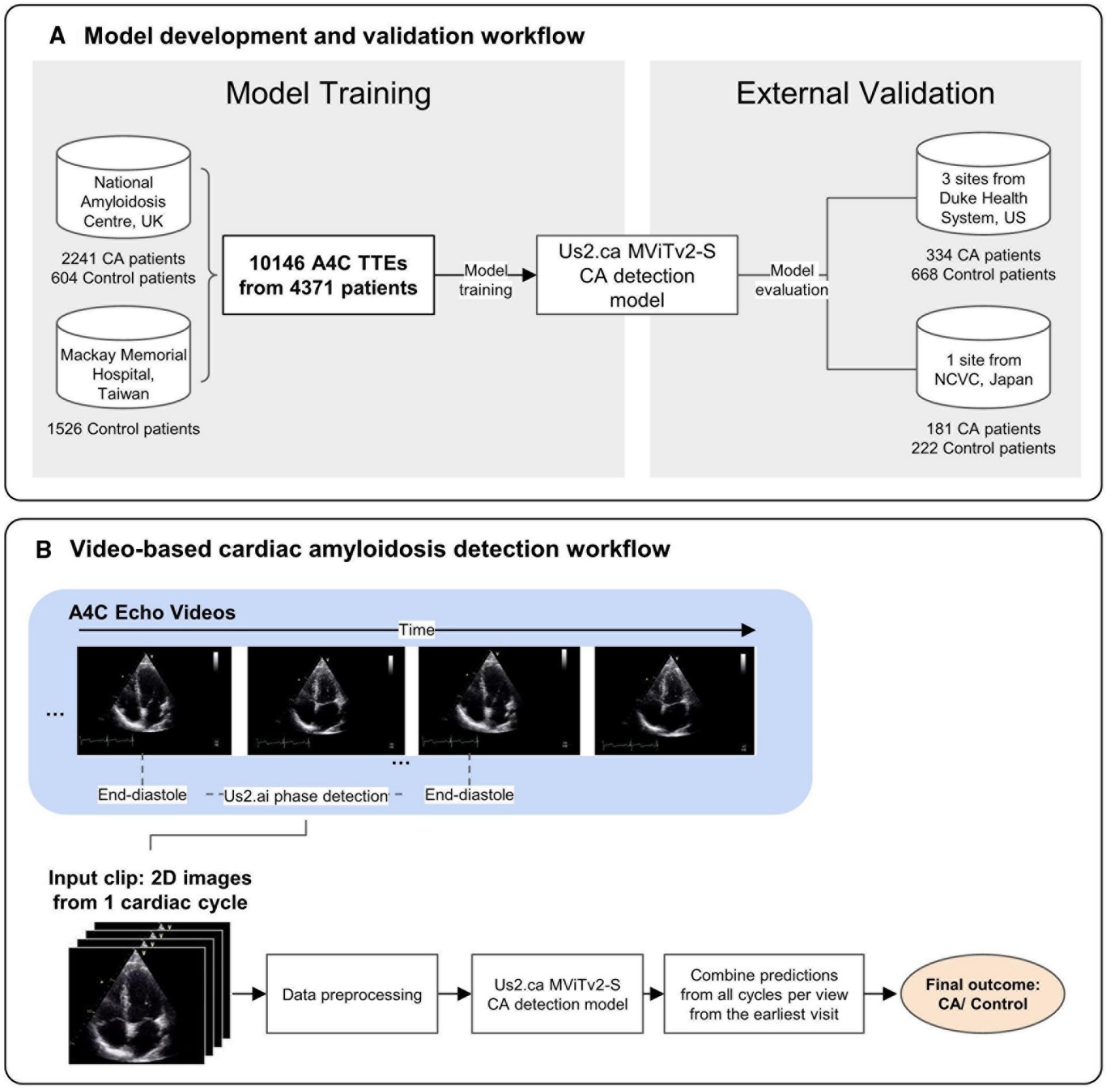
**Training, validation, and functionality of the deep-learning model. A**, Illustration demonstrating how the deep-learning model was trained and validated. **B**, Illustration demonstrating how the deep-learning model determines whether or not a patient has cardiac amyloidosis (CA). A4C indicates apical 4-chamber; NCVC, National Cerebral and Cardiovascular Center; and TTE, transthoracic echocardiogram.

A total of 1069 patients from DUHS and 415 patients from NCVC were initially processed using the Us2.ai software for automated analysis. Patients were excluded if their TTEs lacked an analyzable A4C view or if the available A4C images were of insufficient quality for automated assessment. This process is described in the Supplemental Material. Specifically, 67 DUHS patients (41 suboptimal A4Cs, 26 with no A4C view) and 12 NCVC patients (11 suboptimal A4Cs, 1 with no A4C view) were excluded. These exclusions were necessary to avoid misclassification due to nondiagnostic image quality. The final study cohort, therefore, included 1002 patients from DUHS and 403 patients from NCVC. In total, 515 CA patients and 890 LVH controls were included for external validation (Figure 1A).

#### Ethics

All patients from the NAC provided written consent for their data to be analyzed and published, according to the Declaration of Helsinki and approval from the Royal Free Hospital ethics committee (REC: 21/PR/0620 and 09/H0715/58). The study protocol was reviewed by the institutional review board at all other sites, and a waiver of informed consent was approved to enable retrospective use of deidentified data (TMMH institutional review board number: 20MMHIS370e; DUHS institutional review board number: Pro00115100; NCVC institutional review board number: R24098).

### Multiparametric Echocardiographic Score

The multiparametric echocardiographic score was originally developed using data from NAC and 2 other referral centers in Italy and Spain.^9^ To assess its performance using AI-automated measurements in diverse external data sets, all echocardiograms from the validation cohorts (DUHS, NCVC) were analyzed using a Food and Drug Administration and CE-marked AI software (Us2.ai, Singapore). The software generated fully automated echocardiographic measurements, which were then used to calculate the guideline-based multiparametric echocardiographic score. The score is defined as follows: relative wall thickness >0.6=3 points, E/e’ >11=1 point, TAPSE ≤19 mm=2 points, longitudinal strain ≥−13%=1 point, septal apical-to-base strain ratio >2.9=3 points. Lower scores provide a higher sensitivity, and higher scores provide a higher specificity. A score of ≥6 provides the best balance between sensitivity and specificity, and a score of ≥8 yields the highest specificity.^9^

### Video-Based Deep-Learning CA Detection Model (Us2.ca)

Us2.ca is a video-based deep-learning model developed for automated detection of CA, using the small variant of the Multiscale Vision Transformers architecture.^17^ The model analyzes A4C TTE clips, with each input video representing a single cardiac cycle. Details of data preprocessing are provided in the Supplemental Material.

The Multiscale Vision Transformers architecture divides input videos into spatiotemporal patches, projects them into D-dimensional embeddings, and hierarchically extracts features through progressive pooling layers. It incorporates relative position embeddings to enhance spatial awareness and residual pooling connections to reduce information loss during down-sampling, thereby improving the preservation of critical temporal and spatial features for visual tasks. The small variant reduces computational complexity, enabling faster inference and efficient deployment in the clinical screening of CA.

During training, the Multiscale Vision Transformers architecture model was initialized with pretrained weights from the Kinetics-400 video classification task provided by PyTorch and trained using a cross-entropy loss function and the Adam optimizer. The initial learning rate was set to 1×10^−4^, and a batch size of 5 was used. To facilitate learning, the learning rate decayed exponentially by a factor of 0.98 after each epoch. A stopping criterion was implemented such that training would end if the accuracy metric stopped improving after 5 consecutive epochs. Data augmentation was also randomly applied to improve the model’s generalizability.

The best accuracy on the internal validation set was achieved at the 30th epoch. Model training and validation were completed using Python version 3.11 with Pytorch version 2.1.1 on a workstation fitted with an NVIDIA GeForce RTX 3090 graphics processing unit of 24GB video RAM. The entire training process took ≈29 hours.^17^

### Us2.ca Beat-to-Beat CA Assessment

The Us2.ca model generates a probability score for CA detection for each A4C video clip, with patient-level predictions obtained by averaging the scores across all cardiac cycles and views (Figure 1B). Patients are classified as positive for suspected CA if the patient-level score exceeds an upper cutoff of 0.80 and negative if the score falls below a lower cutoff of 0.45. Scores between these thresholds are considered indeterminate, yielding a <100% yield. These thresholds were selected based on internal validation to balance sensitivity and specificity, achieving the smallest absolute difference between the 2 while maintaining a high yield. The cutoffs were held constant across all external validation data sets. For comparison, the model was also evaluated using a single cutoff of 0.625, corresponding to the midpoint between the earlier 2 thresholds, to allow classification of all indeterminate cases.

### Statistical Analysis

Model performance was evaluated using classification metrics, such as area under the receiver-operating characteristic curve (AUC), sensitivity, specificity, yield, and accuracy. The 95% CIs of AUCs were calculated using the DeLong method, while all other inferential statistics, accompanied by 95% CIs, were calculated using the Wald method.

Model calibration was assessed using predicted probabilities for the positive class. The multiparametric echocardiographic scores were rescaled to the 0 to 1 range to approximate a probability. Calibration was evaluated by grouping predictions into deciles and plotting the observed event rate against the mean predicted value in each bin. A linear regression of observed outcomes on the predicted logit provided the calibration intercept and slope, summarizing agreement between predicted and observed risks.

To assess model generalisability across heterogeneous real-world settings, the DUHS and NCVC cohorts were combined to generate an additional pooled estimate of performance and to ensure adequate sample size for clinically relevant subgroup analyses. Subgroup analyses were exploratory, and pairwise comparisons were performed without formal adjustment for multiplicity.

Unless specified otherwise, statistical tests were 2-sided, with a significance threshold of alpha <0.05. Baseline characteristics were summarized as mean±SD for normally distributed data and as median with interquartile range for non-normally distributed data. Model performance analyses were conducted in R (version 4.4.2), while model calibration and baseline descriptive statistics were generated in Python (version 3.11).

## Results

### Demographic and Clinical Data

The study population comprised 5776 patients (CA, 2756; controls, 3020). The training data set included patients from the UK NAC and TMMH (CA, 2241; controls, 2130). External test data sets were obtained from the US Duke University Health System (CA, 334; LVH controls, 668) and Japan National Cerebral and Cardiovascular Center (CA, 181; LVH controls, 222). The baseline characteristics are summarized in Table 1.

**Table 1.**
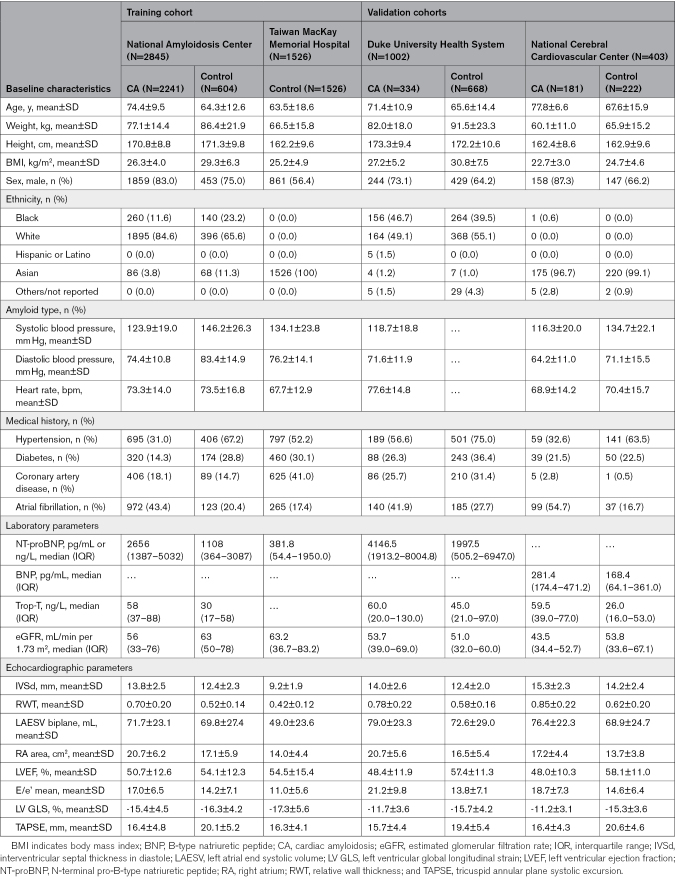
Baseline Characteristics of Patients Included in the Study

### Multiparametric Echocardiographic Score

The multiparametric echocardiographic score for the diagnosis of CA was computed using automated measurements from the Us2.ai software (Figure S1). In the external DUHS population, the score could be calculated in 610 of 1002 patients (60.9%). Cases that could not be classified either had missing images, or the software failed to generate measurements, or had low-confidence measurements resulting from suboptimal image quality (Table S1). Using a threshold of ≥6 points, the score achieved a diagnostic accuracy of 79.5%, sensitivity of 75.4% (95% CI, 69.4%–81.4%), and specificity of 81.5% (95% CI, 77.8%–85.3%). A higher threshold of ≥8 points resulted in increased specificity of 98.1% (95% CI, 96.7%–99.4%) at the cost of sensitivity (27.1%, 95% CI, 21.0%–33.3%), with an overall accuracy of 74.9%. In the NCVC cohort, the score could be calculated in 296 of 403 patients (73.4%). A threshold of ≥6 produced a diagnostic accuracy of 79.7%, sensitivity of 81.6% (95% CI, 75.1%–88.1%), and specificity of 78.1% (95% CI, 71.7%–84.5%). A higher threshold of ≥8 points resulted in a diagnostic accuracy of 69.6%, a sensitivity of 44.1% (95% CI, 35.8%–52.5%), and a specificity of 91.3% (95% CI, 86.9%–95.6%). The calibration slopes were close to 1 (DUHS, 0.94; NCVC, 0.90) and the intercepts near 0 (DUHS, −0.03; NCVC, <0.01), indicating that the score is well-calibrated across both sites. The average end-to-end processing time to generate a full report with the multiparametric score and all the required measurements was 116 seconds per study.

### Video-Based CA Detection Model (Us2.ca)

The Us2.ca model analyzed all A4C clips available in a study to classify patients as having CA or not. In the internal validation cohort, the model yielded confident prediction in 424 of 434 patients (96.6%) and produced a diagnostic accuracy of 96.2%, with a sensitivity of 96.8% (95% CI, 94.4%–99.1%) and specificity of 95.7% (95% CI, 92.9%–98.4%). Similar results were observed in the internal testing cohort, where 431 of 443 patients (97.3%) were confidently classified and produced a diagnostic accuracy of 95.8%, sensitivity of 97.3% (95% CI, 95.1%–99.4%), and specificity of 94.3% (95% CI, 91.2%–97.4%).

**Figure 2.**
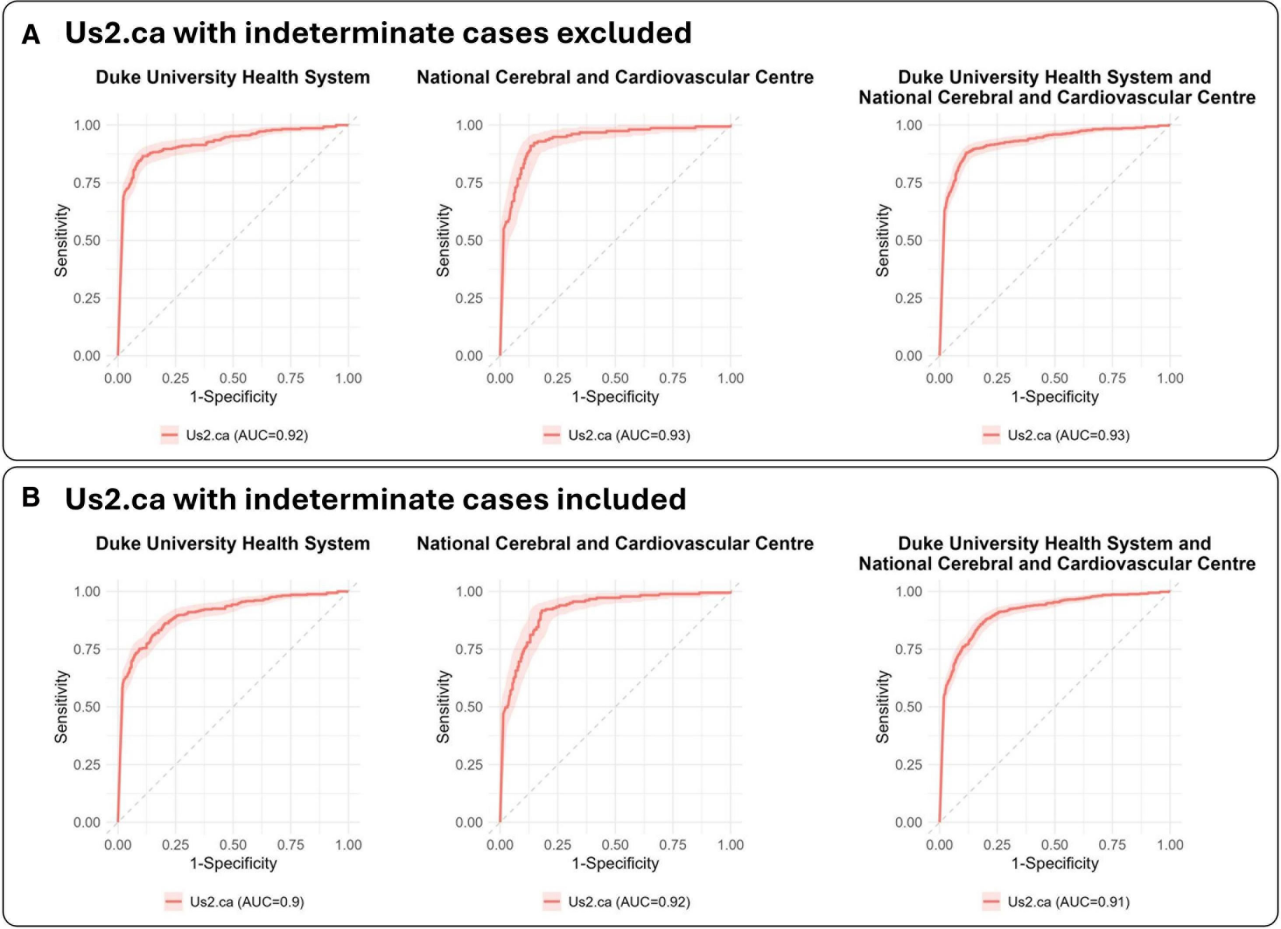
**Area under the receiver-operating characteristic curves (AUC) for the deep-learning model. A**, Receiver-operating characteristic curves demonstrating the Us2.ca model’s performance using 2 thresholds and therefore excluding indeterminate cases. **B**, Receiver-operating characteristic curves demonstrating the Us2.ca model’s performance using 1 threshold, to allow classification of all indeterminate cases.

The Us2.ca model was subsequently tested in the external DUHS data set, and generated confident outputs for 869 of 1002 patients (86.8%), achieving a diagnostic accuracy of 87.5%, sensitivity of 86.6% (95% CI, 82.6%–90.5%), and specificity of 87.9% (95% CI, 85.3%–90.6%). In the external NCVC data set, 352 of 403 patients (87.3%) were confidently classified, obtaining a diagnostic accuracy of 88.4%, sensitivity of 92.3% (95% CI, 88.1%–96.5%), and specificity of 85.3% (95% CI, 80.3%–90.2%). Patients without a confident output were considered indeterminate, resulting in <100% yield (Table 2; Figure 2A). The calibration slopes were <1 (DUHS, 0.68; NCVC, 0.80), suggesting some degree of overconfidence in predicted probabilities, particularly at the DUHS site, but the intercepts were near 0 (DUHS, 0.02; NCVC, <0.01), indicating minimal systematic bias in predictions.

**Table 2.**
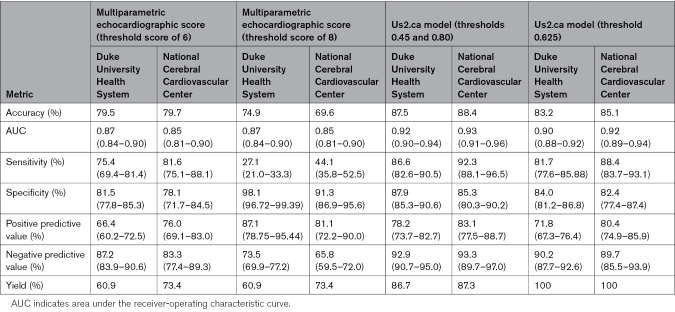
Performance of the Multiparametric Echocardiographic Score and Deep Learning Model in the External Validation Cohorts for Patients With a 4-Chamber View of Adequate Quality for Analysis

The Us2.ca model was also tested using a single threshold, which allowed for the classification of all patients with an adequate A4C view, thereby removing indeterminate results. In the DUHS data set, this approach produced a diagnostic accuracy of 83.2%, a sensitivity of 81.7% (95% CI, 77.6%–85.9%), and a specificity 84.0% (95% CI, 81.2%–86.8%). In the NCVC cohort, the diagnostic accuracy was 85.1%, with a sensitivity of 88.4% (95% CI, 83.7%–93.1%) and a specificity of 82.4% (95% CI, 77.4%–85.9%; Table 2; Figure 2B). The calibration slopes were <1 (DUHS, 0.65; NCVC, 0.81), suggesting some degree of overconfidence in predicted probabilities, but the intercepts were near 0 (DUHS, 0.01; NCVC, <0.01), indicating minimal systematic bias in predictions (Figure S2).

The external DUHS and NCVC cohorts were combined to assess the diagnostic capabilities of Us2.ca across different subgroups. In the combined external data set, the diagnostic performance of Us2.ca was consistent across different vendors (GE versus Philips, AUC 0.94 [95% CI, 0.92–0.97] versus AUC 0.92 [95% CI, 0.90–0.94]; *P*=0.149; Table S2). The diagnostic performance was also consistent across younger and older patients (age <70 years versus age ≥70 years (AUC, 0.91 [95% CI, 0.87–0.94] versus AUC, 0.93 [95% CI, 0.91–0.95]; *P*=0.202) male and female patients (AUC, 0.92 [95% CI, 0.91–0.94] versus AUC, 0.93 [95% CI, 0.90–0.96]; *P*=0.740), White and Black patients (AUC, 0.91 [95% CI, 0.87–0.94] versus AUC, 0.94 [95% CI, 0.91–0.97]; *P*=0.179) and patients with transthyretin CA and light-chain CA (AUC, 0.94 [95% CI, 0.92–0.95] versus AUC, 0.90 [95% CI, 0.87–0.94]; *P*=0.104). The diagnostic performance of Us2.ai across the different subgroups was maintained when a single threshold was used (Table S3).

**Figure 3.**
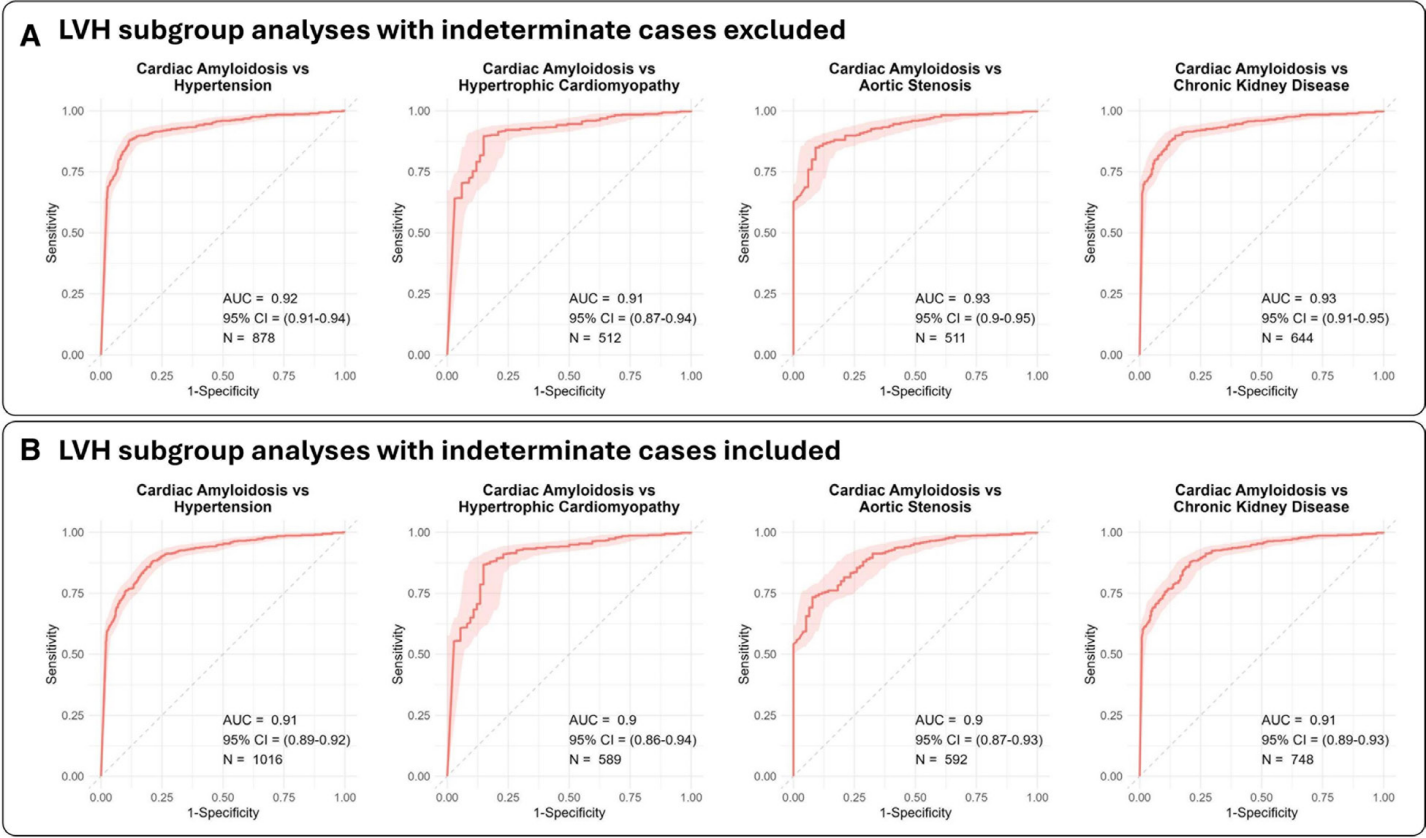
**Area under the receiver-operating characteristic curves (AUC) for the deep-learning model demonstrating its ability to differentiate cardiac amyloidosis from other hypertrophic phenotypes. A**, Receiver-operating characteristic curves demonstrating the Us2.ca model’s performance using 2 thresholds and therefore excluding indeterminate cases. **B**, Receiver-operating characteristic curves demonstrating the Us2.ca model’s performance using 1 threshold, to allow classification of all indeterminate cases. LVH indicates left ventricular hypertrophy.

The model demonstrated robust performance in distinguishing CA from several hypertrophic phenocopies, achieving high AUCs across pairwise comparisons (2 thresholds: CA versus hypertension: AUC, 0.92 [95% CI, 0.91–0.94]; CA versus hypertrophic cardiomyopathy: AUC, 0.91 [95% CI, 0.87–0.94]; CA versus aortic stenosis; AUC, 0.93 [95% CI, 0.90–0.95]; CA versus chronic kidney disease: AUC, 0.93 [95% CI, 0.91–0.95]; 1 threshold: CA versus hypertension, AUC, 0.91 [95% CI, 0.89–0.92]; CA versus hypertrophic cardiomyopathy, AUC, 0.90 [95% CI, 0.86–0.94]; CA versus AS, AUC, 0.90 [95% CI, 0.87–0.93]; CA versus chronic kidney disease: AUC, 0.91 [95% CI, 0.89–0.93]; Figure 3). Importantly, diagnostic accuracy was preserved across the spectrum of LVH, with AUCs of 0.85 (95% CI, 0.80–0.90) for maximal wall thickness (MWT) ≤14 mm, 0.91 (95% CI, 0.86–0.95) for MWT 14 to 16 mm, 0.94 (95% CI, 0.90–0.98) for MWT 16 to 18 mm, 0.93 (95% CI, 0.86–0.99) for MWT 18 to 20 mm, and 0.85 (95% CI, 0.71–1.00) for MWT >20 mm (Figure S3). The diagnostic performance of Us2.ai was maintained across the spectrum of LVH when a single threshold was used (Table S4).

**Figure 4.**
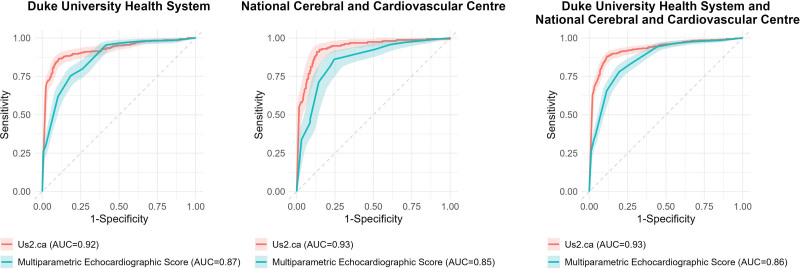
**Comparison of the multiparametric echocardiographic score to the Us2.ca model.** Receiver-operating characteristic curves comparing the performance of the multiparametric echocardiographic score to the Us2.ca model. AUC indicates area under the receiver-operating characteristic curves.

In the subset of patients in whom all the measurements required to calculate the multiparametric echocardiographic score were available and the Us2.ca model provided a confident result, Us2.ca out-performed the multiparametric score in the DUHS population (AUC, 0.93 [95% CI, 0.91–0.96] versus AUC, 0.88 [95% CI, 0.85–0.91]; *P*<0.001), NCVC population (AUC, 0.92 [95% CI, 0.89–0.96] versus AUC, 0.87 [95% CI, 0.83–0.92]; *P*=0.015), and the combined external population (AUC, 0.93 [95% CI, 0.91–0.95] versus AUC, 0.88 [95% CI, 0.85–0.90]; *P*<0.001; Figure 4).

The average analysis time of Us2.ca was 0.83 seconds per video clip, and a report was generated in 68 seconds per study.

## Discussion

This international multicentre study demonstrates that (1) AI-derived echocardiographic measurements can be incorporated into the established multiparametric echocardiographic scoring system to accurately detect CA; (2) an automated AI-based deep-learning model using only single-view (A4C) echocardiographic images can accurately distinguish patients with CA from a wide variety of LVH controls; (3) the deep-learning model was able to classify a greater proportion of patients with a superior diagnostic accuracy compared with the multiparametric echocardiographic score computed from AI-derived measurements. These findings, taken collectively, advance the field for patients and health systems by enabling the automated detection of CA in patients undergoing routine echocardiograms.

The AI-derived multiparametric echocardiographic score was able to accurately differentiate between patients with CA and control patients with LVH across a range of different health care settings. The optimal threshold of ≥6 points provided a high diagnostic accuracy of 79.5% and 79.7% in the external DUHS and NCVC cohorts. AI-derived measurements can be easily incorporated into the scoring system to screen for CA and efficiently refine the diagnostic differentials, without the requirement of any human interaction. However, the diagnostic yield of the multiparametric echocardiographic score is limited by the availability of the measurements required to compute the score, and hence, a proportion of patients could not be classified using this method alone.

To overcome these limitations, a deep-learning model, Us2.ca, was developed and validated. The model underwent robust and comprehensive evaluation, initially with internal validation and testing, and subsequently with external validation in 2 different international populations, with echocardiographic images acquired using multiple different vendors, all of which confirmed a consistent and accurate diagnostic performance. The high diagnostic accuracy (>87%) in diverse external international cohorts and maintenance of model performance across diverse patient subgroups (age, sex, race, and amyloid subtype) are reassuring for the external applicability of our results.

Furthermore, the deep-learning Us2.ca model only requires a single A4C video to classify patients, taking <1 second to analyze each video clip, and hence, was able to classify a greater proportion of patients than the multiparametric scoring system with a greater diagnostic accuracy.^9^ The algorithm can be applied retrospectively to stored echocardiograms to screen for missed cases of CA, or prospectively in real-time image capture to highlight the potential presence of CA in patients undergoing echocardiography. The simplicity of the approach holds promise for broad screening applications in both specialized and nonspecialized clinical care settings, as well as in clinical research and trial settings.

These findings support emerging evidence that AI has the potential to improve echocardiographic detection of CA. Early studies were limited by small sample sizes and, therefore, the performance across different subgroups was either lacking or not formally reported, limiting the generalisability of data and applicability of the results. These studies also lacked a comparison of the model performance to the established multiparametric echocardiographic score, and therefore, the added benefit of the model over standard clinical practice was unclear.^18^ More recently, a large multisite study developed a model to detect CA and compared the model performance to that of the multiparametric echocardiographic score derived from manual measurements.^19^ Although this represents an important comparison, the use of manual measurements introduces significant intraobserver and interobserver variability, which is likely to impact the performance of the echocardiographic score.^13^

The current study represents the first validation of the multiparametric echocardiographic score computed from AI-derived measurements, and the subsequent comparison with a fully automated deep-learning model confirms the added benefit of the deep-learning model with regard to diagnostic yield and accuracy. The increased efficiency of both the AI-derived multiparametric score and the deep-learning model has important implications on workflow. Both techniques could be integrated into clinical practice as fully automated background screening tools that are applied without any human interaction to echocardiograms of patients with heart failure symptoms to help raise the suspicion of CA. Such an approach has the potential to identify possible cases of CA, which may have otherwise been overlooked, and prompt a referral to a specialist center for confirmatory diagnostic tests.

### Limitations

This study is retrospective, and therefore these results require validation in prospective studies. The TMMH population provided the majority of training controls and was labeled solely on echocardiography imaging criteria, rather than explicit screening for CA. However, the model performed similarly during external validation and, therefore, is unlikely to have been affected by the lack of explicit screening. The diagnostic algorithms were tested in high-prevalence populations and therefore require validation in low-prevalence populations where the pretest probability of CA would also be reduced. The multiparametric echocardiographic score could not classify a subset of patients due to the limited availability of necessary measurements, and this could introduce a selection bias.

### Conclusions

In summary, both the multiparametric echocardiographic score computed from AI-derived measurements and the fully automated deep-learning model can accurately identify patients with CA across a range of health care settings. Both techniques represent useful diagnostic tools that could be easily incorporated into clinical practice to help raise the suspicion of CA and provide clinicians with a selection of strategies that could be used in combination to enhance confidence in clinical decision-making.

## Article Information

### Disclosures

Dr Fontana is supported by a British Heart Foundation Intermediate Clinical Research Fellowship (FS/18/21/33447) and has consulting income from Intellia, Novo-Nordisk, Pfizer, Eidos, Prothena, Akcea, Alnylam, Caleum, Alexion, Janssen, Ionis, and AstraZeneca. Dr Gilmore has consulting income from Ionis, Eidos, Intellia, Alnylam, and Pfizer. Dr Wechalekar has consulting income from Alexia, AstraZeneca, Janssen, Attralus, and Prothena. Dr Solomon has received research grants from Alexion, Alnylam, AstraZeneca, Bellerophon, Bayer, BMS, Cytokinetics, Eidos, Gossamer, GSK, Ionis, Lilly, MyoKardia, NIH/NHLBI, Novartis, NovoNordisk, Respicardia, Sanofi Pasteur, Theracos, US2.AI, and has consulted for Abbott, Action, Akros, Alexion, Alnylam, Amgen, Arena, AstraZeneca, Bayer, Boeringer-Ingelheim, BMS, Cardior, Cardurion, Corvia, Cytokinetics, Daiichi-Sankyo, GSK, Lilly, Merck, Myokardia, Novartis, Roche, Theracos, Quantum Genomics, Cardurion, Janssen, Cardiac Dimensions, Tenaya, Sanofi-Pasteur, Dinaqor, Tremeau, CellProThera, Moderna, American Regent, Sarepta, Lexicon, Anacardio, Akros, Valo. A. Ioannou has received consulting fees from AstraZeneca, Bayer, and Prothena and speaker fees from Alexion, AstraZeneca, and Pfizer. Dr Kouri has acted as a consultant, advisor, or speaker for Alnylam Pharmaceuticals, AstraZeneca, BridgeBio Pharma (formerly Eidos Therapeutics), and Pfizer. S.C. Lim is employed by Us2ai and owns stock options in Us2ai. M. Frost is employed by Us2ai and owns stock options in Us2ai. J. Ezekowitz reports grants and personal fees from Amgen, grants and personal fees from Bayer, grants and personal fees from American Regent, grants and personal fees from Merck, grants and personal fees from Otsuka, grants and personal fees from Novo Nordisk, grants and personal fees from Applied Therapeutics, grants and personal fees from Cardurion, grants and personal fees from CSL-Vifor, grants and personal fees from AstraZeneca, grants and personal fees from BI-Lilly, grants and personal fees from US2.ai, during the conduct of the study. Dr Lam is supported by a Clinician Scientist Award from the National Medical Research Council of Singapore; has received research support from NovoNordisk and Roche Diagnostics; has served as consultant or on the Advisory Board/Steering Committee/Executive Committee for Alnylam Pharma, AnaCardio AB, Applied Therapeutics, AstraZeneca, Bayer, Biopeutics, Boehringer Ingelheim, Boston Scientific, Bristol Myers Squibb, Corteria, CPC Clinical Research, Eli Lilly, Impulse Dynamics, Intellia Therapeutics, Ionis Pharmaceutical, Janssen Research & Development LLC, Medscape/WebMD Global LLC, Merck, Novartis, Novo Nordisk, Quidel Corporation, Radcliffe Group Ltd., Roche and Us2.ai; and serves as Cofounder & nonexecutive director of Us2.ai and has the following patents: (1) patent pending: PCT/SG2016/050217, method for diagnosis and prognosis of chronic heart failure, licensor: Agency for Science, Technology and Research, Singapore; National University of Singapore; National University Hospital Singapore Pte Ltd. (2) US Patent No. 10,702, 247, automated clinical workflow that recognizes and analyzes 2-dimensional and Doppler echo images for cardiac measurements and the diagnosis, prediction, and prognosis of heart disease, licensor: Us2.ai. (3) Proposed SG patent application, multi-marker panel to distinguish between heart failure with preserved versus reduced ejection fraction, Applicants: NUS; NUH; A*STAR, A*STAR ref: DxD/P/12220/00/SG; IP2020-400-01, NUS Ref: 2020-400-01, NUHS ref: NUHSRO-ID/2020/021, MIRXES ref: TBA. The other authors report no conflicts.

### Supplemental Material

Supplemental Methods

Tables S1–S4

Figures S1–S3

## Supplementary Material
