# Supplementary material for: Diagnosis of Cardiac Amyloidosis on Echocardiography Using Artificial Intelligence
Source: Circ Cardiovasc Imaging. 2026 Feb 12;19(4):e018991. doi: 10.1161/CIRCIMAGING.125.018991 (PMC13095060; doi:10.1161/CIRCIMAGING.125.018991)
Supplement: Supplementary file 1 [file hci-19-e018991-s001.pdf]

Supplementary material

Data pre-processing

Echocardiogram studies from CA and Control cases, consisting of videos and images (e.g., Doppler imaging) in the standardized Digital Imaging and Communications in Medicine (DICOM) format, undergo pre-processing before being used as inputs for the Us2.ca model during both training and testing. This media undergoes processing by the Us2.ai software, which classifies the echocardiographic views, and identifies diastolic and systolic frames. A4C videos are selected, and utilizing the diastolic and systolic frames, they are divided into clips representing individual cardiac cycles.

The A4C video clips were deemed to be of sufficient quality for analysis using an automated deep-learning view-classifier model that is part of the FDA-approved and CE-cleared Us2.ai software, which has been previously validated.[11] The software automatically identifies the echocardiographic view based on the highest output probability generated by the classifier. Studies were excluded when the probability score indicating the presence of an A4C view fell below the predefined threshold. By relying on a fixed, quantifiable output from a validated automated tool, this approach removes inter-reader variability and ensures that the exclusion process is fully reproducible.

This approach also addresses potential variability in image quality across the study period and across different institutions. All echocardiograms regardless of acquisition year or institution underwent the same automated view selection and quality assessment. This standardized approach ensures that all included A4C clips meet a uniform quality threshold, mitigating variability due to differences in acquisition time periods.

Using Us2.ai automated processing pipeline, the selected A4C clips are cropped to remove background pixel data around the echocardiogram, resized to 224x224 pixels per frame, and

underwent gray-scaling, and uniform resampling to a fixed length of 16 frames. The resultant A4C video clips serve as inputs to the Us2.ca model.

Us2.ai software was used solely to ingest DICOM images and automatically identify A4C views. All model development was performed outside the software environment. During training, images were extracted and pre-processed using custom Python scripts, after which the deep-learning model was trained entirely in Python. During testing, the final trained model was integrated into the Us2.ai software to enable a fully automated prediction pipeline with the same pre-processing code used in training. The software generated the model's predictions, which were then exported for downstream analysis.

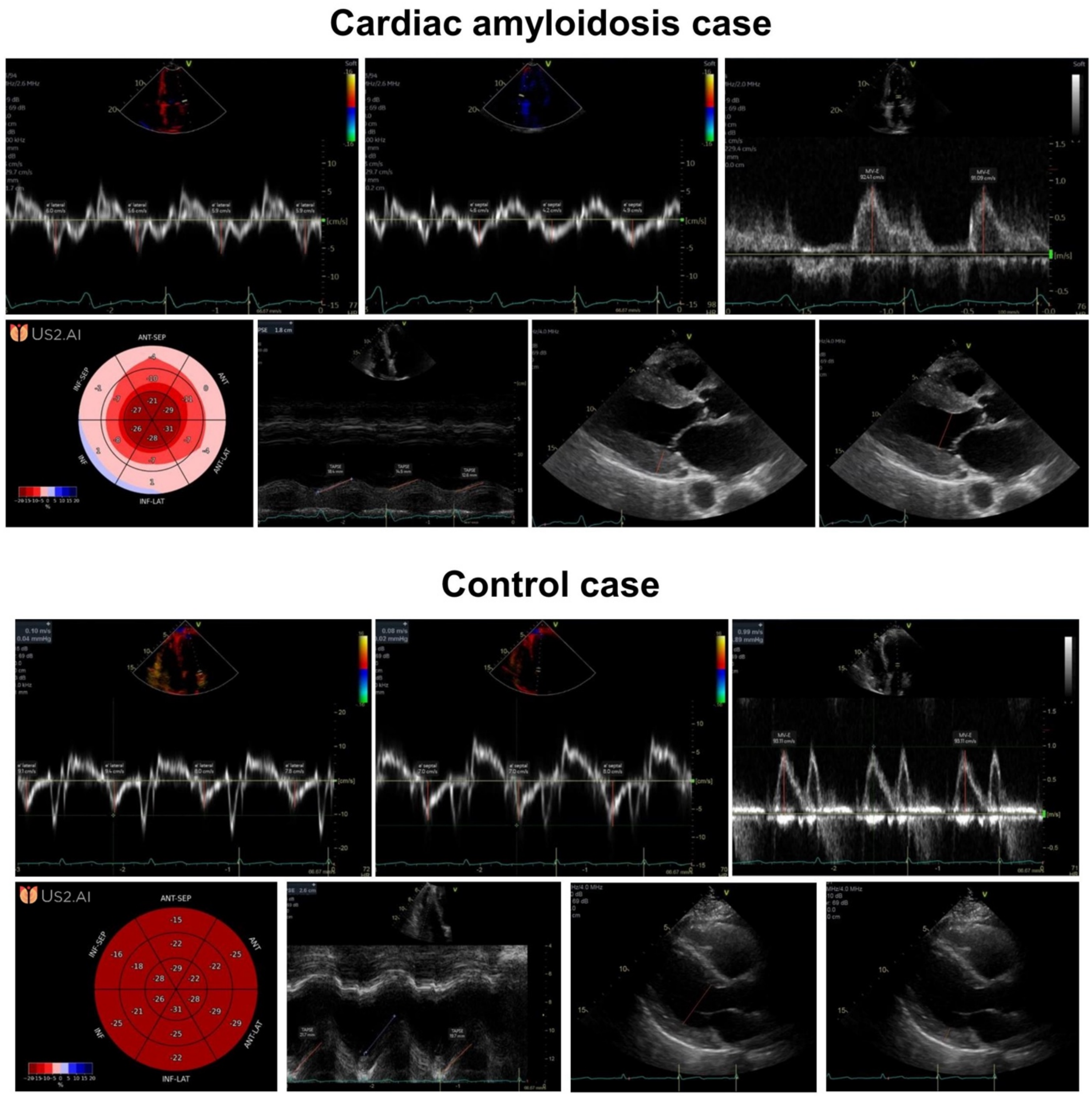

Supplementary Figure S1. Examples of artificial intelligence derived measurements being used to calculate the multiparametric echocardiographic score in patients with cardiac amyloidosis and controls.

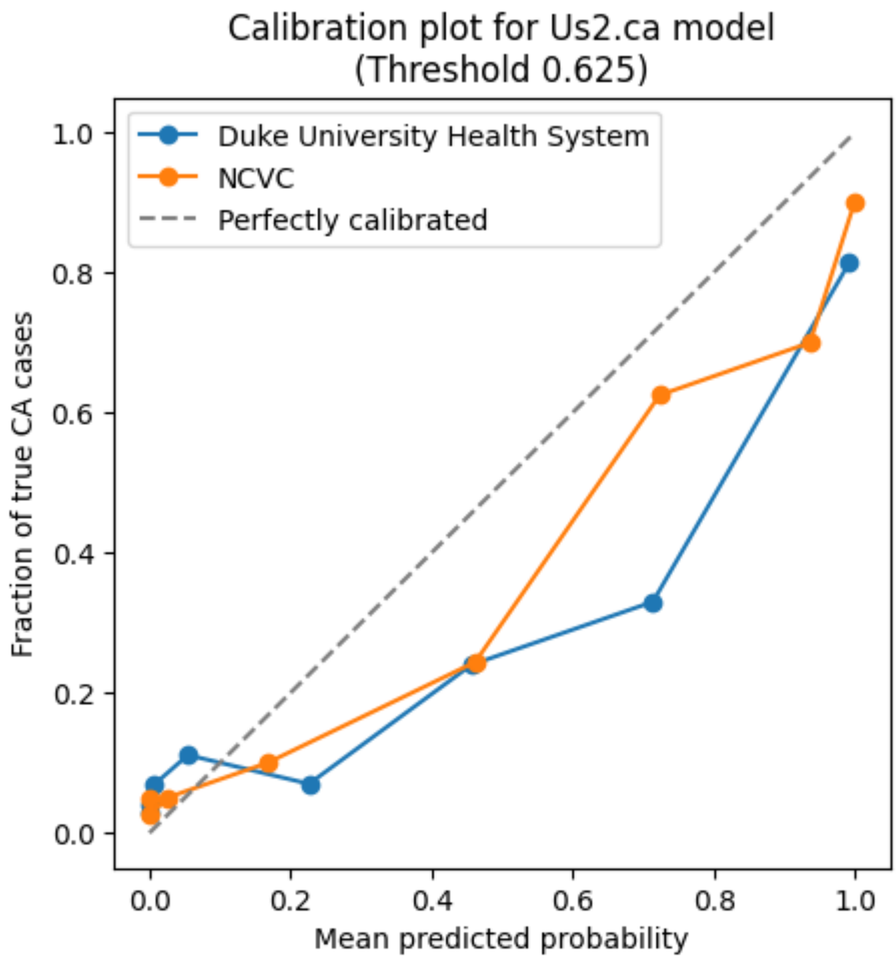

Supplementary Figure S2. Calibration curves for DUHS and NCVC using the Us2.ca model at a single cut-off of 0.625. NCVC = National Cerebral and Cardiovascular Centre

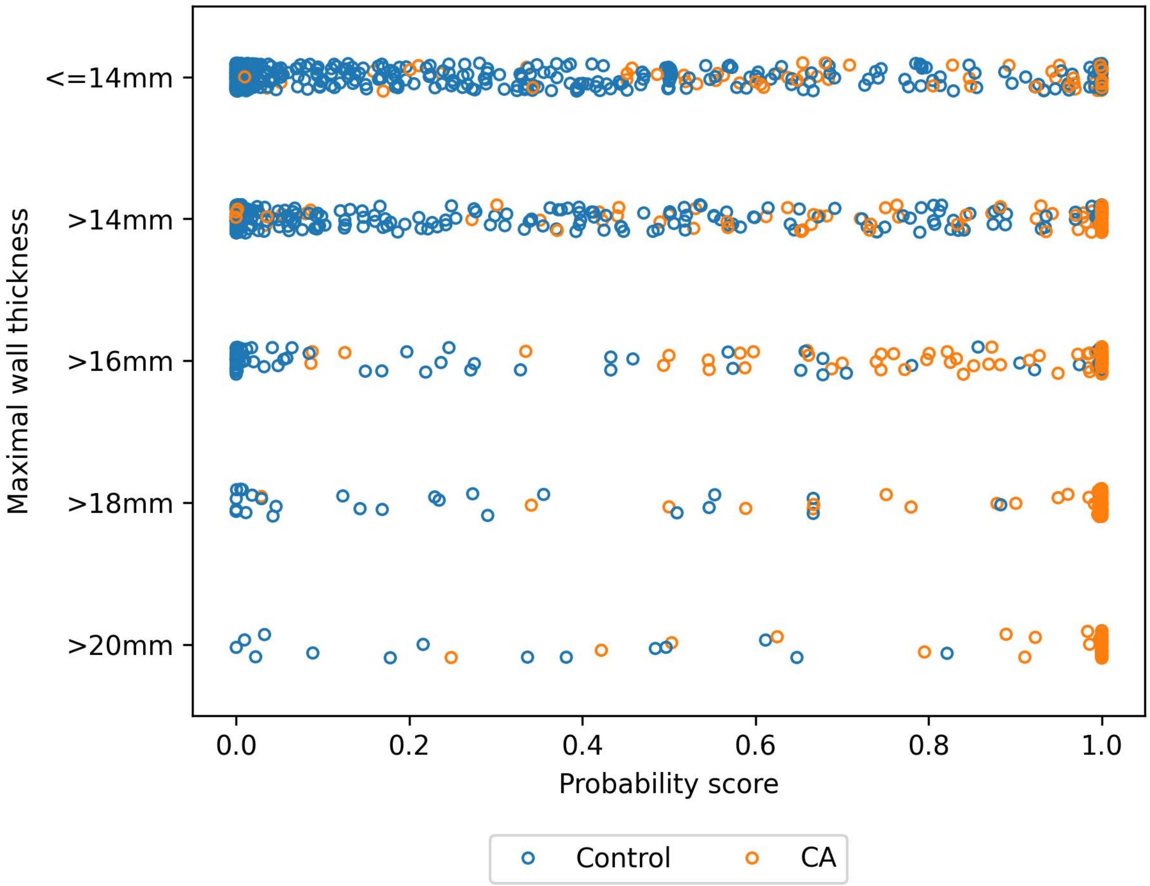

Supplementary Figure S3. Plot demonstrating the probability scores generated by the Us2.ai model across the spectrum of left ventricular hypertrophy. CA = Cardiac amyloidosis, MWT = Maximal wall thickness

Measurement	N patients with missing images (%)		N patients where Us2.ai failed to generate a measurement or reported low confidence (%)	
	DUHS (N=1002)	NCVC (N=403)	DUHS (N=1002)	NCVC (N=403)
RWT	0 (0%)	0 (0%)	34 (3.39%)	1 (0.25%)
E/e'	97 (9.68%)	33 (8.19%)	138 (13.77%)	47 (11.66%)
TAPSE	161 (16.07%)	32 (7.94%)	62 (6.19%)	6 (1.49%)
LS and SAB	0 (0%)	0 (0%)	17 (1.70%)	1 (0.25%)

Supplementary Table S1. Measurements missing from the multiparametric echocardiographic score. "N patients with missing images" refers to cases where the relevant view for the respective echocardiographic measurement was not available. "N patients where Us2.ai failed to generate a measurement or reported low confidence" refers to cases where the software was unable to produce a reliable measurement. DUHS = Duke University Health System; NCVC = National Cerebral and Cardiovascular Centre; LS = Longitudinal strain; RWT = Relative wall thickness; SAB = Septal apical-to-base strain ratio; TAPSE = Tricuspid annular plane systolic excursion;

**Subgroup Comparison of Us2.ca (Thresholds 0.45 and 0.80)**	**GE (N=719)**	**Philips (N=685)**
Accuracy (%)	88.3	87.1
Area under curve (AUC)	0.94 (0.92-0.97)	0.92 (0.90-0.94)
Sensitivity (%)	93.1 (89.4, 96.9)	85.5 (81.3, 89.7)
Specificity (%)	86.4 (83.2, 89.6)	88.38 (84.9, 91.9)
Positive predictive value (%)	72.8 (66.9, 78.6)	85.8 (81.6, 90.0)
Negative predictive value (%)	97.0 (95.3, 98.7)	88.1 (84.6, 91.6)
Yield (%)	86.8%	87.0%
AUC comparison p-value	0.149	

Supplementary Table S2. Diagnostic performance of the Us2.ca model across different vendors.

Only one patient in the combined external cohort had missing vendor details.

Subgroup Comparison	Us2.ca model (Thresholds 0.45 and 0.80)			Us2.ca model (Threshold 0.625)		
	AUC - Group 1	AUC - Group 2	p-value	AUC - Group 1	AUC - Group 2	p-value
Age <70 vs ≥70	0.91 (0.87-0.94)	0.93 (0.91-0.95)	0.202	0.89 (0.86-0.93)	0.91 (0.89-0.93)	0.358
Male vs Female	0.92 (0.91-0.94)	0.93 (0.90-0.96)	0.740	0.91 (0.89-0.93)	0.92 (0.88-0.95)	0.666
White vs Black	0.91 (0.87-0.94)	0.94 (0.91-0.97)	0.179	0.89 (0.86-0.92)	0.92 (0.89-0.95)	0.147
ATTR vs AL	0.94 (0.92-0.95)	0.90 (0.87-0.94)	0.104	0.92 (0.90-0.94)	0.88 (0.85-0.92)	0.057

Supplementary Table S3. Diagnostic performance of the Us2.ca model across different subgroups. Group 1 refers to the subgroup on the left and group 2 refers to the subgroup on the right.

Subgroup Analyses	AUC of Us2.ca model (Thresholds 0.45 and 0.80)	AUC of Us2.ca model (Threshold 0.625)
Maximal wall thickness ≤14mm	0.85 (0.80-0.90)	0.84 (0.79-0.88)
Maximal wall thickness 14-16mm	0.91 (0.86-0.95)	0.88 (0.84-0.92)
Maximal wall thickness 16-18mm	0.94 (0.90-0.98)	0.92 (0.88-0.96)
Maximal wall thickness 18-20mm	0.93 (0.86-0.99)	0.92 (0.86-0.98)
Maximal wall thickness > 20mm	0.85 (0.71-1.00)	0.87 (0.76-0.99)

Supplementary Table S4: Diagnostic performance of the Us2.ca model across different wall thicknesses.
